# Differential Encoding of Factors Influencing Predicted Reward Value in Monkey Rostral Anterior Cingulate Cortex

**DOI:** 10.1371/journal.pone.0030190

**Published:** 2012-01-18

**Authors:** Koji Toda, Yasuko Sugase-Miyamoto, Takashi Mizuhiki, Kiyonori Inaba, Barry J. Richmond, Munetaka Shidara

**Affiliations:** 1 Doctoral Program in Kansei, Behavioral and Brain Sciences, Graduate School of Comprehensive Human Sciences, University of Tsukuba, Tsukuba, Ibaraki, Japan; 2 Human Technology Research Institute, National Institute of Advanced Industrial Science and Technology, Tsukuba, Ibaraki, Japan; 3 Faculty of Medicine, University of Tsukuba, Tsukuba, Ibaraki, Japan; 4 Laboratory of Neuropsychology, National Institute of Mental Health, National Institutes of Health, Department of Health and Human Services, Bethesda, Maryland, United States of America; University of California Davis, United States of America

## Abstract

**Background:**

The value of a predicted reward can be estimated based on the conjunction of both the intrinsic reward value and the length of time to obtain it. The question we addressed is how the two aspects, reward size and proximity to reward, influence the responses of neurons in rostral anterior cingulate cortex (rACC), a brain region thought to play an important role in reward processing.

**Methods and Findings:**

We recorded from single neurons while two monkeys performed a multi-trial reward schedule task. The monkeys performed 1–4 sequential color discrimination trials to obtain a reward of 1–3 liquid drops. There were two task conditions, a valid cue condition, where the number of trials and reward amount were associated with visual cues, and a random cue condition, where the cue was picked from the cue set at random. In the valid cue condition, the neuronal firing is strongly modulated by the predicted reward proximity during the trials. Information about the predicted reward amount is almost absent at those times. In substantial subpopulations, the neuronal responses decreased or increased gradually through schedule progress to the predicted outcome. These two gradually modulating signals could be used to calculate the effect of time on the perception of reward value. In the random cue condition, little information about the reward proximity or reward amount is encoded during the course of the trial before reward delivery, but when the reward is actually delivered the responses reflect both the reward proximity and reward amount.

**Conclusions:**

Our results suggest that the rACC neurons encode information about reward proximity and amount in a manner that is dependent on utility of reward information. The manner in which the information is represented could be used in the moment-to-moment calculation of the effect of time and amount on predicted outcome value.

## Introduction

A great deal of evidence suggests that the anterior cingulate cortex (ACC) relates reward to motivation, cognition, and action [1]–[3]. Anatomical experiments show that there are dense connections between the ACC and reward-related brain areas, such as midbrain dopamine neurons [4]–[6] and limbic regions [7], [8], whose neurons respond to value of the reward [9]–[14]. Neurons in the ACC are known to respond to reward and error events [15], [16], reward prediction errors [17], reward expectancy [18], [19], reinforcement learning [20], reward-based action selection [21], decision making [22]–[29], and fictive reward learning [30], [31]. All of these suggest that the ACC has a role in processing information about reward value.

The subjective value of the reward is influenced by intrinsic reward value and the length of time to obtain it. We previously reported that the caudal ACC plays a role in long-term reward expectancy, that is, the neuronal response is modulated by the reward proximity in a schedule of trials [18]. It has also been reported that neuronal activity in the ACC is modulated by the expected reward amount [16], [23], [25]–[31]. To investigate whether the rostral ACC (rACC) neurons differentially encode the reward proximity and reward amount information, we recorded from single neurons in monkey rACC while manipulating the reward proximity and amount trial-by-trial in a reward schedule task. We used a modified version of a reward schedule task we have used previously [18], in which the monkeys performed schedules of one to four sequential color discrimination trials to earn one to three drops of reward. We examined the responses of rACC neurons under two task conditions, one where the number of trials and reward amount were related to a visual cue that made information available to the monkeys about the reward schedule and the upcoming reward amount, i.e., a valid cue condition, and the other where the cue was picked at random, i.e., a random cue condition.

## Materials and Methods

### Ethics Statement

The experiments were carried out with two adult male rhesus monkeys (*Macaca mulatta*) weighing 7–9 kg. All experiments were approved by the Animal Care and Use Committee of the National Institute of Advanced Industrial Science and Technology (AIST) (permission number: 32-06-013 and 32-07-013) and the Animal Care and Use Committee of University of Tsukuba (permission number: 08-124, 09-190, and 10-080), and were performed in strict accordance with the Guideline for Care and Use of Animals of AIST and the Guideline for Care and Use of Animals of University of Tsukuba. These guidelines are based on the recommendations of the National Research Council (USA) as published in the ILAR “Guide for the Care and Use of Laboratory Animals”, and all research procedures followed the recommendations of the ILAR Guide, therefore also consistent with the recommendations of the Weatherall Report on “The Use of Non-Human Primates in Research”.

### Experimental conditions

Monkeys squatted in a standard primate chair and faced a 20 inch cathode-ray tube (CRT) monitor (FlexScan E66T; Eizo Nanao, Ishikawa, Japan) placed 95 cm in front of them. A touch sensitive bar was attached to the front panel of the primate chair at the level of the monkey's hand. Water was delivered from a tube positioned in front of the monkey's mouth as a reward. The only light in the testing room came from the CRT monitor. Real-time experimental control and data acquisition were performed using the REX program adapted for the QNX operating system [32]. Neurobehavioral Systems Presentation software was used to display visual stimuli (Neurobehavioral Systems, Inc., Albany, CA).

### Behavioral Procedures

The behavioral paradigms and visual stimuli used in the present study were designed based on a previously used reward schedule task [33]–[35]. In the version used here, two factors, the schedule length (1, 2, 3, or 4 trials to earn the reward for Monkey T; 1, 2, or 3 trials for Monkey I) and the reward amount (1, 2, or 3 drops of water for both monkeys), were manipulated independently in a crossed design.

**Figure 1:**
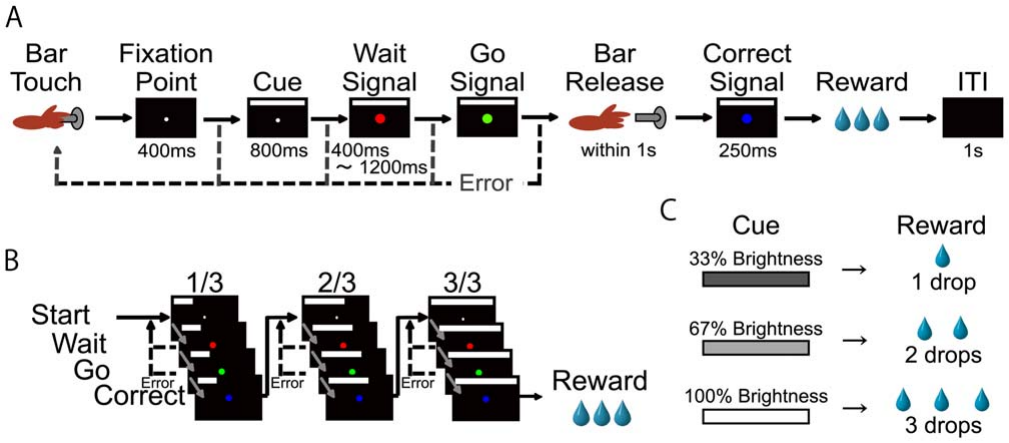
Behavioral task. A, Time sequence of task events in an individual color discrimination trial in the rewarded trial with 3 drops of reward in the valid cue condition. B, Example of the sequence in 3-trial schedule with 3 drops in the valid cue condition. The visual cue was presented on the top of the screen. Length of the visual cue indicates schedule state (remaining trials to earn the reward; 1, 2, or 3 trials). C, Brightness of the visual cue indicates reward amount (1, 2, or 3 drops of water).

Both monkeys were first trained to perform a sequential color discrimination task (Fig. 1A). In the color discrimination task, the monkey touched the bar in the chair to initiate a trial. A small white square, 0.17×0.17 deg, appeared immediately on the center of the screen. After 400 milliseconds a visual cue, 24×0.6 deg appeared at the top of the screen. After another 800 ms, the fixation point was replaced with a 0.4×0.4 deg red visual target (Wait signal). Then, after a randomly chosen wait time (400, 600, 800, 1000, or 1200 ms), the 0.4×0.4 deg visual target turned green (Go signal). Finally, if the monkey released the touch-bar within 1 s after the visual target turned green, the visual target turned blue for 250 ms (Correct signal) and then disappeared. An error was counted when the monkey released the touch-bar either when the bar was released too early (earlier than 150 ms after the onset of the Go signal), or when the monkey failed to release the bar before the Go signal disappeared. When the monkey made these bar release errors, the visual cue and visual targets were extinguished and the trial was terminated immediately. The intertrial interval (ITI) was 1 s after a correct trial and 2 s after an error. When the monkey completed more than 80% of trials correctly for two consecutive training days, the reward schedule task was introduced (Monkey T with 1-, 2-, 3-, and 4-trial schedules, Monkey I with 1-, 2-, and 3-trial schedules). In the reward schedule task, the monkey was required to complete randomly chosen schedules of one, two, three, or four trials of the sequential color discrimination. The monkeys had to complete each schedule before beginning a new one, no matter how many errors were made. After an error trial, the monkey had to repeat the same trial with same cue and reward condition until the trial was completed correctly. The reward was delivered after a correct response in the last trial of the schedule. On correct trials in which no reward was delivered, a reward apparatus with the delivery valve turned off was activated (sham reward). The visual cue brightened as the trial progressed to the rewarded trial. The brightness of the visual cue in each trial was proportional to the schedule state, i.e.,
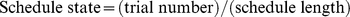
(i.e., 1/1 for 1-trial schedule; 1/2, and 2/2 for 2-trial schedule; 1/3, 2/3, and 3/3 for 3-trial schedule; 1/4, 2/4, 3/4, and 4/4 for 4-trial schedule)

After the performance in the reward schedule task stabilized, another factor, reward size was added so that there were three possible levels of reward amount (1 drop, 2 drops, and 3 drops of water; approximately 0.15, 0.30, and 0.45 ml respectively). In the reward-schedule-amount-task, the brightness and length of the single visual cue indicated the reward amount and schedule, respectively (Fig. 1B and 1C). The brightness of the visual cue was proportionally related to the reward amount, i.e., pixel intensities in eight bit; 85 (33.3%) for 1 drop, 170 (66.6%) for 2 drops, and 255 (100%, 30.19 lux) for 3 drops. The visual cue lengthened as the trial progressed to the rewarded trial, i.e., 1/4, 25% of full length (6.06×0.60 deg); 1/3, 33.3% of full length (8.08×0.60 deg); 1/2 and 2/4, 50% of full length (12.12×0.60 deg); 2/3, 66.6% of full length (16.16×0.60 deg); 3/4, 75% of full length (18.18×0.60 deg); 1/1, 2/2, 3/3, and 4/4, 100% of full length (24.24×0.60 deg).

Two task conditions were used, valid cue and random cue conditions. In the valid cue condition, length and brightness of the visual cue indicated reward proximity and amount, respectively. In the random cue condition, the schedule sequence and the manipulation of the reward amount remained, but the length and the brightness of the visual cue was chosen randomly from the cue set. After an error trial in the random cue condition, the same visual cue was presented until the trial was completed correctly. In most recording sessions, during the search for a single unit, the monkey was performing in the valid cue condition. Valid cue and random cue conditions were run in blocks (with each block generally having more than 300 trials) and were changed without signaling the change of the condition.

### Surgery

After the monkeys were trained to perform the reward-schedule-amount task, a sterile surgical procedure was carried out under general anesthesia to place a recording chamber and a head holder (Crist Instrument Co., Inc., Hagerstown, MD). Before the surgery, a magnetic resonance image (MRI) at 3T was obtained. The center of the recording chamber was fixed in the stereotaxic plane centered at 33 mm rostral to the interaural line (A 33) and 4 mm lateral to the midline (L 4) based on the MRI.

Surgery was carried out in a dedicated sterile operating room using sterile procedures under Ketamine and Pentobarbital anesthesia. The monkeys received antibiotics for 1 week after the surgery to reduce the risk of postoperative infections and were given a 2 week postoperative recovery period. The postoperative recovery was uneventful. The post-operative animal was carefully observed for signs that may indicate pain or distress. The monkeys showed no such symptoms after the surgery. Neuronal recordings began immediately after the recovery period of the surgery.

### Single-unit recording

Single-unit activity was recorded while the monkeys performed the reward-schedule-amount task. All the neurons were tested in the valid cue condition first, followed by the random cue condition if the single unit remained well isolated. A hydraulic microdrive (MO-97A Oil Hydraulic Micromanipulator, Narishige, Tokyo, Japan) was mounted on the recording chamber, and tungsten microelectrodes (impedance: 1.0–1.4 MΩ; Micro Probes, Inc, MD) were inserted vertically through a stainless steel guide tube that was placed in a hole of a grid (Crist Instrument Co., Inc.) within the recording chamber. Single-unit activity was isolated using spike sorter (Sankei Co., ltd, Tokyo, Japan), where unit isolation was performed on-line by principal component analysis [36], [37].

An MRI was acquired with a tungsten microelectrode inserted to confirm the recording location [38].

### Data analysis

All data analyses were performed in the R statistical computing environment (R Development Core Team, 2004).

To examine the effects of two reward value parameters, i.e., reward schedule and reward amount, on behavioral performance of the monkey, the percentage of errors was examined. The percentage of errors was calculated for each experimental condition, and was defined as percentage of the number of error trials per the total number of trials in each schedule state and reward amount across all the recording sessions, resulting in a single grand percentage of errors for each schedule state and reward amount in both valid and random cue condition. Statistical significance was tested using the chi-squared test (*p*<0.05).

For neuronal activity, we first tested whether or not each neuron responded to task events. To calculate a baseline activity, we compared number of spikes within a 400-ms period before the fixation point appearance in the first trial of each schedule (1/1, 1/2, 1/3, and 1/4) and the number of spikes in the non-first trial (2/2, 2/3, 3/3, 2/4, 2/4, 3/4, and 4/4), and adopted the smaller one of the two. We then tested whether or not a neuron showed significant change in activity comparing to the baseline activity during the following 8 task events: (1) a 400-ms period after the fixation point appearance (“fixation” period), (2) a 400-ms period after the appearance of the cue (“cue” period), (3) a 400-ms period after the “wait” signal onset (“wait” period), (4) a 400-ms period after the “go” signal onset (“go” period), (5) a 400-ms period around the timing of the bar-release (from −200 to 200 ms after the bar-release, “bar-release” period), (6) a 400-ms period after the “ok” signal onset (“ok” period), (7) a 400-ms period around the deactivation of reward apparatus in last drop (including the sham and reward valve) (from −200 to 200 ms after the deactivation of reward apparatus in last drop, “reward” period), (8) a 400 ms period from 200 ms to 600 ms after last reward drop (“ITI” period). There were 10 schedule states and 8 task events for t-test analysis; the significance level was corrected to be 0.000625 according to the Bonferroni method. If the result of the t-test was significant for at least 1 out of 8 task events, the neuron was counted as an event-related neuron.

To test whether the activity of the event-related neurons showed modulations depending on the reward schedule or reward amount, we categorized schedule state as 3 levels: 1/2, 1/3, and 1/4 for “first trial level”, 2/3, 2/4, and 3/4 for “intermediate trial level”, and 2/2, 3/3, and 4/4 for “rewarded trial level” because the responses in each level appeared to be similar. Since we were interested in studying how multiple trials to earn the reward affects neuronal activity in the rACC, the 1/1 trials was eliminated from the analysis. We analyzed the neuronal responses using two-way ANOVA (schedule state 3 levels: first, intermediate and rewarded trial, reward amount 3 levels: small, medium, and large; *p*<0.01). For the schedule state level, there were 2 two-way ANOVA models: (1) 9 levels of schedule states where the levels coded whether the trial was categorized for each schedule state, i.e., 1/2, 2/2, 1/3, 2/3, 3/3, 1/4, 2/4, 3/4, and 4/4 schedule state, and (2) 3 levels of schedule levels where the levels coded whether the trial was categorized for 3 schedule progress level, i.e., first (1/2, 1/3, and 1/4), intermediate (2/3, 2/4, and 3/4), and rewarded trial (2/2, 3/3, and 4/4). To evaluate which of the two ANOVA models was better for analyzing the neuronal data, we compared 9-level ANOVA model and 3-level ANOVA model by F-test using the “anova” function in R. The null hypothesis of the F-test for model comparison was that the variances of errors between actual values and expected values calculated from each model were equal [39]. This procedure determines whether the extra degrees of freedom in the 9-level ANOVA are justified. If the difference was not significant, the simpler model, i.e., the 3-level ANOVA, was the preferred model for neuronal data. We found that 67.6% of the task-related neurons (194/287) showed no significant differences, so we adopted the 3-level ANOVA model for all the data. To quantify the degree to which neuronal activity depended upon the factors of interest, we collated the percentage of the variance in the neuronal activity explained by each factor. This measure is related to calculating the power of the neuronal signal for each factor [40]. After the ANOVA analysis, if the schedule state factor was significant (*p*<0.01), we tested each pair (first-intermediate, intermediate-rewarded, and first-rewarded) *post-hoc* using the Tukey honest significant difference (Tukey HSD) test (*p*<0.05). We classified specific schedule level selective neuron as follows. (1) If there was a significant difference between first and intermediate, and between first and rewarded, but not between intermediate and rewarded, we classified the neuron as first-selective neuron. (2) If there was a significant difference between first and intermediate, and between intermediate and rewarded, but not between first and rewarded, we classified the neuron as intermediate-selective neuron. (3) If there was a significant difference between first and rewarded, and between intermediate and rewarded, but not between first and intermediate, we classified the neuron as reward-selective neuron. If the reward amount factor was significant (*p*<0.01), we tested each pair (small-medium, medium-large, and small-large) using the Tukey HSD test (*p*<0.05). We classified reward amount selective neurons in a manner similar to that above as follows. (1) If there was significant difference between small and medium, and between small and large, but not between medium and large, we classified the neuron as small reward-selective neuron. (2) If there was significant difference between small and medium, and between medium and large, but not between small and large, we classified the neuron as medium reward-selective neuron. (3) If there was significant difference between small and large, and between medium and large, but not between small and medium, we classified the neuron as large reward-selective neuron.

We also examined whether the neuronal response showed graded modulation through schedule progress. Because graded modulation can be observed during the 4-trial and 3-trial schedule, we examined the neuronal response during 4-trial and 3-trial schedule (one-way ANOVA with the four or schedule state levels, i.e., 1/4, 2/4, 3/4, and 4/4 for the data from monkey T, and 1/3, 2/3, and 3/3 for the data from monkey I). If the schedule state factor was significant (*p*<0.01), we tested each pair of 4 schedule states (1/4-2/4, 1/4-3/4, 1/4-4/4, 2/4-3/4, 2/4-4/4, and 3/4-4/4) or 3 schedule states (1/3-2/3, 1/3-3/3, and 2/3-3/3) using *post-hoc* Tukey HSD test (*p*<0.05). If the post hoc analysis revealed a significant difference (Tukey HSD test, *p*<0.05), the strength of the activity in each pair was compared by calculating the averaged spike counts. We categorized the neuron as decreasing type neuron when the strength of the activity was 1/4>2/4>3/4>4/4, or 1/4>2/4>3/4≒4/4, or 1/4>2/4≒3/4>4/4, or 1/4≒2/4>3/4>4/4 for 4-trial schedule, and 1/3>2/3>3/3 for 3-trial schedule. We categorized the neuron as increasing type I neuron when the strength of the activity was 1/4<2/4<3/4<4/4, or 1/4<2/4<3/4≒4/4, or 1/4<2/4≒3/4<4/4, or 1/4≒2/4<3/4<4/4 for 4-trial schedule and 1/3<2/3<3/3 for 3-trial schedule. We categorized the neuron as increasing type II neuron when the strength of the activity was 1/4<2/4<3/4, and 4/4<3/4 for 4-trial schedule and 1/3<2/3 and 3/3<2/3 for 3-trial schedule. We analyzed the peak response for total population, and then analyzed the response in each task event.

## Results

### Behavioral data

**Figure 2:**
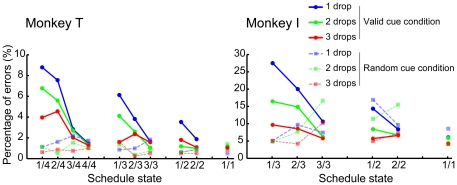
Percentage of errors in the task. Solid lines show the percentage of errors in the valid cue condition. Broken lines show the percentage of errors in the random cue condition. Percentage of errors (%) is shown on the ordinate and schedule state on the abscissa. Reward amount is shown by line color (1 drop, blue; 2 drops, green; and 3 drops, red). Percentage of errors was calculated as the total number of errors divided by the total number of trials (×100) in each schedule state and reward amount across all recording sessions (Monkey T: 233 sessions in the valid cue condition and 75 sessions in the random cue condition, Monkey I: 75 sessions in the valid cue condition and 10 sessions in the random cue condition.

For both monkeys, the percentage of errors decreased significantly with schedule progress (chi-squared test, *p*<0.05) and with increasing reward amount (chi-squared test, *p*<0.05) (Fig. 2, solid lines) in the valid cue condition. The percentage of errors was small and indistinguishable across all schedule states (chi-squared test, *p*>0.05) and all reward amounts (chi-squared test, *p*>0.05) (Fig. 2, broken lines) in the random cue condition. These results show that the monkeys were sensitive to information provided by the visual cue (schedule progress and reward amount) in the valid cue condition, and that the monkeys were not sensitive to the cue when the information about reward amount and schedule state was not provided in the random cue condition.

### Neuronal data

**Figure 3:**
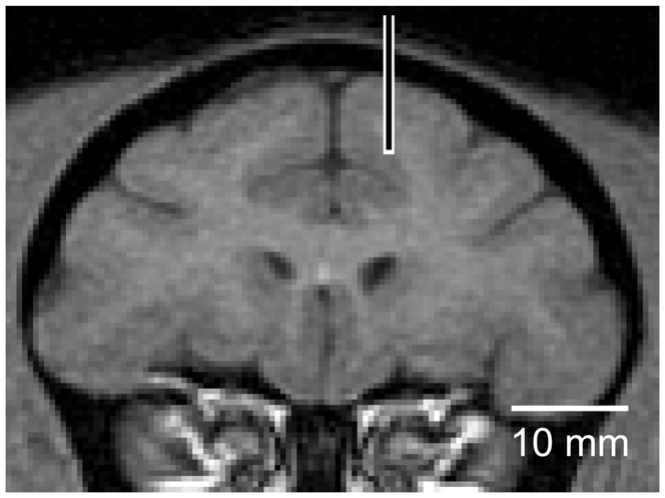
Recording site. MR image (coronal section at anterior 33 to the interaural line ; monkey T) is shown. An example of electrode position is shown. MRIs were obtained on a 3-T General Electric Sigma unit.

We recorded from 308 neurons in three hemispheres of the two monkeys (233 neurons in monkey T, 75 neurons in monkey I). Using MRI we confirmed that all of the recorded neurons were located in either the dorsal or ventral bank of the rACC (A30 to A39; an example is shown in Fig. 3). All 308 neurons were tested in the valid cue condition, and 85 neurons were also tested during the random cue condition (75 neurons from monkey T, 10 neurons from monkey I).

Of the 308 neurons, 287 (93.2%) neurons showed significant task-related activity in the valid cue condition and 75/85 (88.2%) neurons showed task-related activity in the random cue condition (t-test with Bonferroni correction, *p*<0.000625). The percentage of neurons responding for each task event is shown in Table 1.

**Table 1:** Percentage of neurons showing significant task-related activity.

	Events							
Condition	Precue	Cue	Wait	Go	Release	OK	Reward	ITI
Valid cue (N = 308)	56.2%	74.7%	73.1%	71.4%	70.1%	69.8%	57.1%	53.6%
Random cue (N = 85)	35.3%	57.6%	60.0%	65.9%	70.6%	69.4%	65.9%	62.4%

#### Graded activity proportional to schedule progress

**Figure 4:**
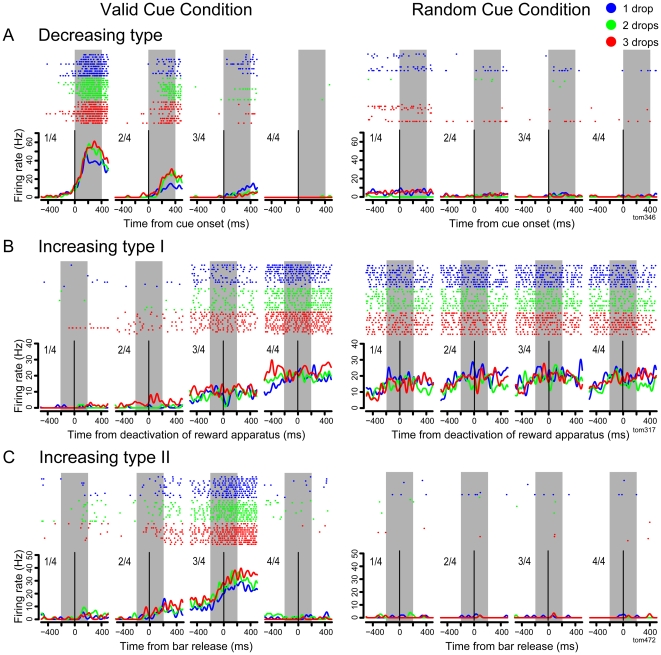
Responses of example rACC neurons: rasters and spike density plots. Three neurons showing the response modulation in relation to the reward schedule progress. The valid cue condition is on the left, and the random cue condition on the right. A, Decreasing activity. Raster and spike density plots are aligned on the time of cue onset (0 ms, vertical line). B, Increasing type I activity (largest response at the rewarded trial), aligning on the deactivation of reward apparatus in the last drop. C, Increasing type II activity (no response at the rewarded trial), aligning on the bar release event. The rasters are classified based on the reward amount, and plotted in the order of trials. The abscissa is time (ms). Colors code for the reward amount. The line plots below the raster are spike density plots. They represent the average spike rate through time across the trials (after smoothing with a 25 ms Gaussian pulse). The ordinate for the spike density plots is firing rate in spikes per second. The gray rectangle in each panel shows the 400 ms window in which the trial-by-trial spikes were counted for statistical analysis in Fig. 5.

**Figure 5:**
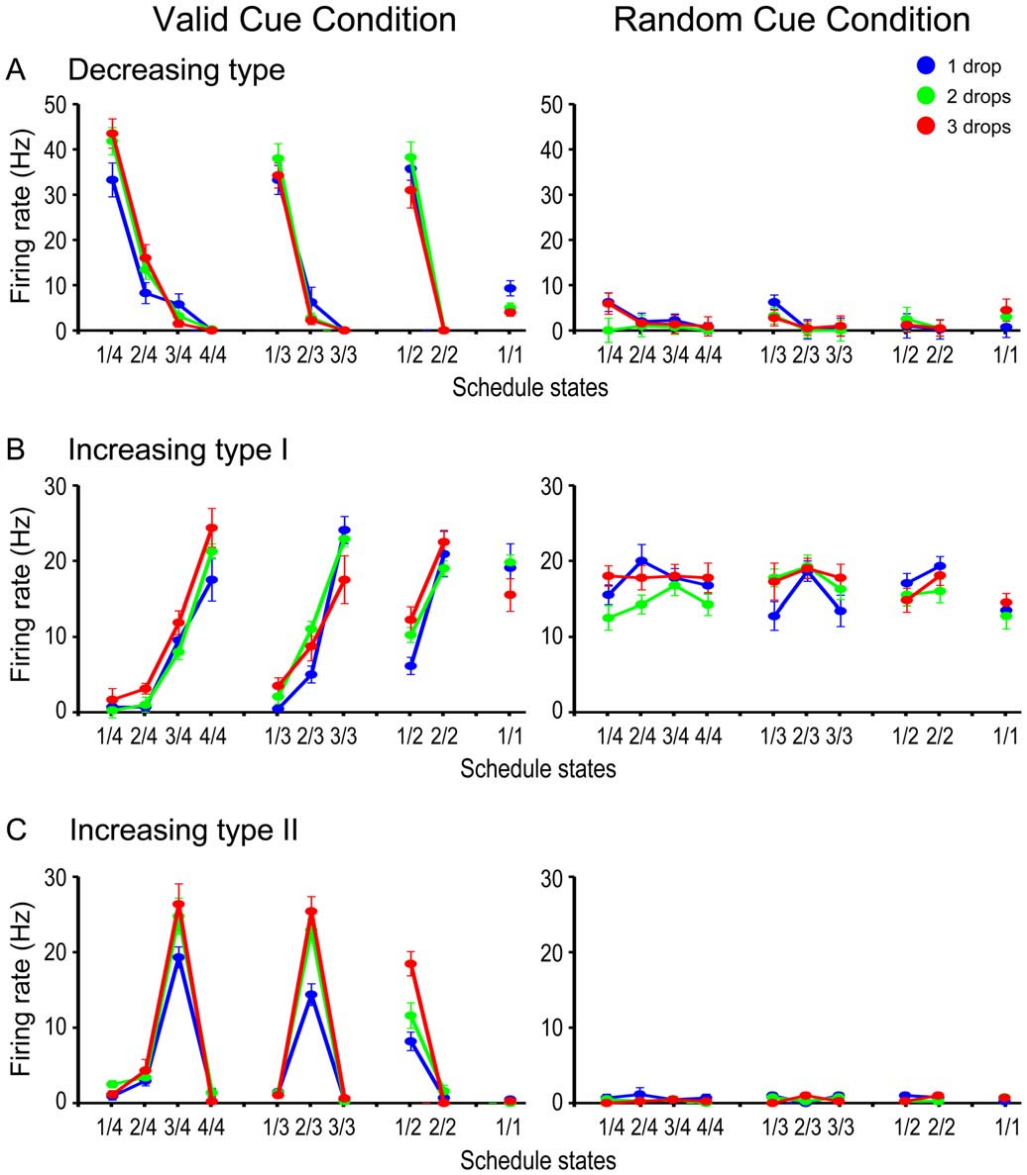
Average firing rate of rACC neurons in **Fig. 4**. Colors mean reward amount as in Figure 4. A, Decreasing type activity. B, Increasing type I activity. C, Increasing type II activity.

**Figure 6:**
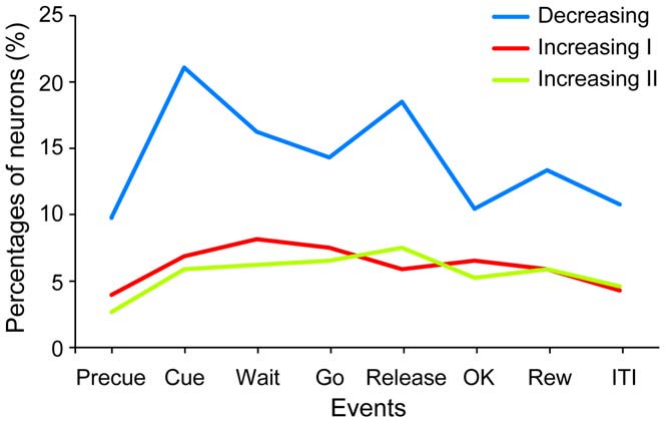
Percentage of neurons that showed graded activity in each task event. Percentage of the decreasing type neurons (blue), increasing type I neurons (red), and increasing type II neurons (green), is shown (N = 308). Percentage of decreasing type neurons was larger than that of increasing type I and II neurons.

Many neurons showed graded activity along with schedule progress, which seems similar to neurons in the caudal part of ACC [18]. To characterize the graded modulation more closely, we analyzed the neuronal data using 4-trial (monkey 1) and 3-trial schedule (monkey 2; N = 308). 57% of the analyzed neurons (174/308) showed graded activity that was directly related to schedule progress in the valid cue condition (Fig. 4). These neurons fell into 3 groups: (1) 105/308 (34.1%) showed “decreasing” activity, where the largest activity was observed in the first trial of each schedule, and the activity decreased along with the schedule progress in the valid cue condition (example Figure 4A). This neuron did not respond in the random cue condition. There was no modulation according to the reward amount (two-way ANOVA, *p*>0.05). Figure 5A shows the mean spike counts in all schedule states for the neuron in Figure 4A. The same trend is observed for all multi-trial schedules. (2) 31/308 (10.1%) showed what we term “increasing type I” activity, where the activity increased along with the schedule progress, with the largest activity in the rewarded trial (example, Figure 4B). This neuron had the same level of activity in all conditions in the random cue condition (two-way ANOVA, *p*>0.05). There was no modulation according to the reward amount. Figure 5B shows the mean spike counts in all schedule states for the same neuron. (3) 38/308 (12.3%) showed what we term “increasing type II“ activity, where the activity increased along with the schedule progress with the largest activity in the trial immediately before rewarded trial (example Figure 4C). This neuron did not respond in the random cue condition, but this neuron did show modulation according to the reward amount (two-way ANOVA, *p*<0.05). Figure 6 shows the percentages of neurons that showed graded activity in each task event (N = 308). The largest proportion showed the decreasing type activity (9.7–21.1%) with the next most frequent showing increasing type I activity (3.9–8.1%) and the least frequent being increasing type II activity (2.6–7.5%). The graded activity observed in the valid cue condition disappeared or lost modulation for all the examined neurons in the random cue condition (52/52, 100%).

#### Effects of schedule state and reward amount in all population

**Figure 7:**
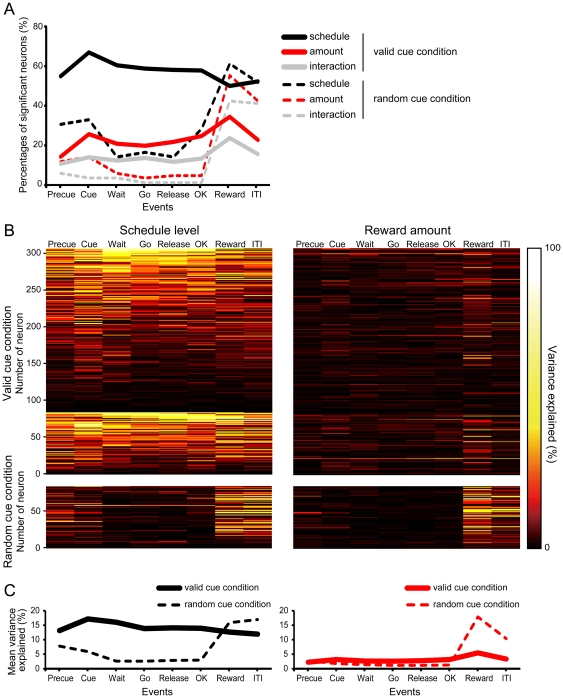
Population results of reward proximity and amount effect. A, Percentages of neurons that showed significant main effect of schedule level and reward amount, and interaction by ANOVA are shown. Black lines show the percentages of neurons that showed schedule level effect, red lines show reward amount effect, gray lines show interaction. Solid lines show the valid cue condition; broken lines for the random cue condition. B, Dynamics of encoding of the schedule state and the reward amount as revealed by the percentage of variance explained for each single neuron. Each line represents the percentage of variance explained in a color heat scale for both valid (upper) and random (lower) cue conditions. The data for each of the 85 (numbered 1 to 85) neurons are the data that were recorded both in the valid and random cue conditions. Neurons are sorted from top to bottom according to the number of events related to a significant response and total value of variance explained of the schedule state, therefore the order of the individual neurons is different in the left and right panels. C, Mean value of valiance explained is shown (summed from data in panel B). Same convention on color as in panel A.

Out of 308 neurons recorded in the valid cue condition, 50.0–66.9% showed schedule level dependent activity through the 8 task events (Fig. 7A, black solid line). A significantly smaller proportion of neurons (14.3–34.4%) showed reward amount dependent activity (Fig. 7A, red solid line), and an even smaller proportion (10.7–23.7%) showed a significant interaction between the schedule level and reward amount (Fig. 7A, gray solid line) (two-way ANOVA, *p*<0.01). We also checked reward amount dependent activity in the first trials only because those trials were where reward amount effect was largest in behavioral data. The percentage of neurons that was sensitive to reward amount was also small (2.0–26.0%).

In the random cue condition, the effect of the schedule level and reward amount in the “reward-expectancy” period (from precue period to ok period) was considerably different from that in the activity in the “reward-delivery” period (reward period and ITI period). In “reward-expectancy” period, the schedule level or reward amount appeared to have a significant effect only in a few neurons (Fig. 7A, dashed lines). In the “reward-delivery” period, however, the percentage of neurons with schedule level or reward amount dependent activity jumped, and reward amount dependent activity became even larger compared with that in the valid cue condition (chi-squared test, *p*<0.05). Of 85 neurons recorded in the random condition, 50 (58.8%) showed reward amount dependent activity in the reward period in the random cue condition. About half of these neurons (26/50) did not show reward amount dependent activity in the reward period in the valid cue condition. However, 24 out of the 26 neurons showed reward amount dependent activity in other events in the valid cue condition.

Another way to look at the effect of the reward proximity and reward amount is to compare the strength of the signals related to these. Figure 7B shows the variance explained by each factor in each task event for each neuron, and Figure 7C shows its average. In the valid cue condition, the response variance explained by the schedule level was significantly greater than that by reward amount through all the task events (Fig. 7C, solid lines; t-test, *p*<0.05). In the random cue condition, the response variance explained by the schedule level was high only in reward and ITI period (Fig. 7C left, dashed line). The response variance explained by the reward amount was also high only in reward and ITI period, which was significantly greater than that in the valid cue condition (Fig. 7C right, dashed line; t-test, *p*<0.05).

#### Neurons encoding specific schedule level or reward amount

**Figure 8:**
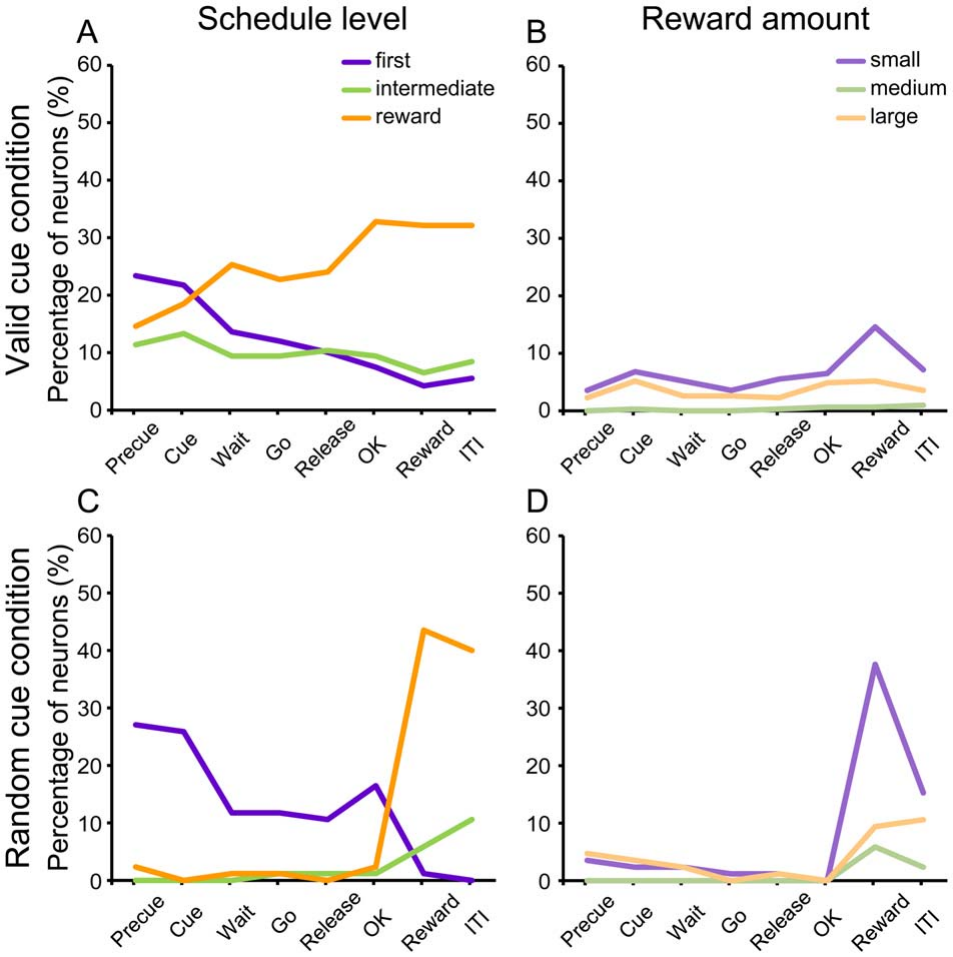
Percentage of neurons encoding information about specific schedule levels or reward amount. Percentage of neurons whose responses in the first trials were larger than those in intermediate and rewarded (purple), whose responses in the intermediate trials were larger than either in the first and rewarded (green), or whose responses in the rewarded trials were larger than in the first and intermediate (orange), is shown in A and C (N = 308). Percentage of neurons whose responses in the small reward amount trials were larger than in the medium and large (purple), whose responses in the medium reward amount trials were larger than either in the small and large (green), or whose responses in the large trials were larger than in the small and medium (orange), is shown in B and D (N = 308). A & B, the valid cue condition. C & D, the random cue condition.

There were also neurons that responded at specific schedule level (first, intermediate, or rewarded trials) or specific reward amount (small, medium, or large). To analyze such idiosyncratic activity, we ranked the neuronal response in each level using a Tukey HSD test (N = 308). Figure 8 shows the percentage of neurons encoding specific schedule level or reward amount in each task event. In the valid cue condition, neurons that were sensitive to each of these levels were observed early in trials, with the largest number for the neurons sensitive to the first trials (Fig. 8A, purple line). As the trial progressed, the responses distinguishing the rewarded trials from all the others rose slightly while the responses distinguishing whether the trials were a first trial declined. The percentage of neurons discriminating the reward amount was smaller (Fig. 8B). In the random cue condition, the percentage of the neurons that discriminated first from other trials was largest in the precue and cue period, and decreased as the trial progressed (Fig. 8C, purple line). And finally, the percentage of neurons discriminating the rewarded vs other trials jumped dramatically upon reward delivery, and remained high in the ITI. This effect probably continues into the next trial, giving rise to the first effect (purple line), because the first trial of one schedule is the trial after a reward in the previous schedule. The largest reward amount effect in the random cue condition is seen for the small reward amount at the time of reward delivery (Fig. 8D).

## Discussion

We simultaneously manipulated reward proximity and reward amount, two factors that affect how a reward is perceived, that is, the subjective outcome, to examine how rACC neurons encode these two factors. Over 90% of the recorded neurons showed some selective activity in the reward-schedule-amount task. These rACC neurons show different responses about rewards in relation to task context, i.e., valid and random cue conditions. When the cue is providing information about reward proximity and reward amount in the valid cue condition, information about predicted reward proximity is represented strongly whereas information about the predicted reward amount is essentially absent at those times. When the cue does not provide information in the random cue condition, as expected, little information about reward proximity or reward amount was encoded during the course of the trial before reward delivery. However, when the information becomes available, that is, when the reward is actually delivered, the number of neurons and the size of the signal for reward proximity and amount become substantial. Thus, the context on reward information determines what kind of and when information is represented in the neuronal firing within the rACC.

In other studies of ACC neurons [16], [25]–[31], there are a substantial number of neurons that show significant modulation related to the reward amount, both predictive and reactive, that is, both predicting the reward in the pre-reward part of the trial, and reflecting the reward that has just been delivered. In our study, there is at best a weak predictive signal about reward amount. Perhaps this difference reflects the difference between animals expecting change in reward amount only vs expecting changes in both reward proximity and reward amount. If the monkeys found the number of trials needed to obtain a reward more salient than reward amount, perhaps that would be the signal that is emphasized in rACC. The reactive signal, that is, the signal when the reward is delivered, carries information about both the reward proximity and reward amount. In the random cue condition, the responses reflect the amount of reward that is actually delivered and the schedule that is just being completed, and, unsurprisingly, this information only appears when the reward is delivered.

The results described just above can be interpreted in light of the difference in reward predictability in two task contexts. In the valid cue condition, subjects are provided with and presumably come to expect reward information at the beginning of the first trial of the schedule. In the random cue condition, the only time the subject receives information about the reward is when the reward is delivered. Thus, these neurons modulate their firing from the earliest point when there is information about the outcome value. In the population the activity provides the signals needed to calculate the value, but there does not appear an explicit signal about the predicted outcome value. Our results suggest that rACC encode just information that can be used to calculate the predicted outcome value in given situation.

### Neurons with graded modulation

The neurons showing progressively decreasing or increasing responses are similar to those seen by Shidara and Richmond [18], even though their recordings were taken from a more caudal location (A19–28) in the anterior cingulate (cACC) compared to the present study (rACC; A30–38). Hayden et al. [29] also reported that neurons in the ACC (A25–30) shows increasing activity along with cumulative time spent selecting same option in a relatively natural task that is directly modeled on real-world foraging situations. Our findings are also quite similar to one aspect of the study by Procyk et al. [41], where they found neurons that they interpreted as keeping track of behavioral sequences, that is, the activity increased or decreased as the monkey worked through a sequence of operant trials representing a learned spatial sequence. The increasing and decreasing neurons could provide the same information about progress through the schedules, so from one point of view they could be considered to be indistinguishable. However, the two groups of neurons might have different functionality. The decreasing type neurons might carry information that is well-suited for keeping track of the current progress from the beginning of the schedule. The increasing type I neurons might carry information that is well-suited for evaluating reward expectancy before reward delivery. The increasing type II neurons might carry information that is well-suited for recognizing the cost that has been irrevocably incurred up to the current time (sunk cost). These signals can be combined to calculate the effect of the passage of time on the predicted outcome value; that is, how much time has passed before a reward will be delivered (temporal discounting), how much time is necessary for earning the reward (reward expectancy), and how much investment this has already cost (sunk cost). Combining the activities of decreasing and increasing neurons in the ACC can provide a means to calculate the temporal discounting with ‘sunk cost’ that added value that accrues from work or time already invested, and is routinely seen in the behavior of the reward schedule task [42].

### Interaction with connected brain areas

The signals we have found could, as described above, be used to compute the time and sequence, thus contributing to calculate outcome value. The ACC has reciprocal connections with the orbitofrontal cortex (OFC) and lateral prefrontal cortex (LPFC) [8], [43]–[45]. Both of these brain regions would seem to be reasonable candidates for incorporating the information from the ACC into their functions because both OFC and LPFC have important roles in reward value encoding [46]–[54]. Reward proximity information from the rACC and information about delay-to-reward and reward amount from the OFC could be integrated in LPFC to modulate the cognitive control signal underlying motor commands [55].

Two other brain regions that might utilize information about predicted reward value originating in ACC are perirhinal cortex [56], [57] and amygdala [7], [8]. When these areas were examined using the reward schedule task [58], [59], neurons in these areas showed cue-related activity. In the perirhinal cortex, only cue-related activity was observed, and the majority of these showed idiosyncratic response to the schedule progress, that is, the neurons responded in an specific set of the trials. In amygdala, neurons showed responses before the cue presentation, before the bar-release, and to the reward delivery as well as to the cue, and cue-related neurons were modulated mainly by the first and rewarded trials of the schedule. The response characteristics of idiosyncratic neurons in rACC overlap the neurons in the amygdala. Although it is not clear how these might influence one another, the rACC and the amygdala may have important role in associating the visual cue with reward information.

### Conclusion

Our results suggest that the rACC neurons encode information about reward proximity and amount in a context dependent manner. When the cue is providing information about reward, information about predicted reward proximity is more strongly represented than information about predicted reward amount. When the cue does not provide information, the information about reward amount was largely confined to the period when the reward is delivered. The manner in which the information is represented in both gradually decreasing or increasing responses as the trials progress through the reward schedules provide signals that could be used in the moment-to-moment calculation of the effect of waiting time on predicted outcome value.
